# Impact of Activity-Based Working and Height-Adjustable Desks on Physical Activity, Sedentary Behavior, and Space Utilization among Office Workers: A Natural Experiment

**DOI:** 10.3390/ijerph17010236

**Published:** 2019-12-28

**Authors:** Takashi Jindo, Yuko Kai, Naruki Kitano, Kyohsuke Wakaba, Mitsuru Makishima, Koji Takeda, Michitaka Iida, Kinji Igarashi, Takashi Arao

**Affiliations:** 1 Physical Fitness Research Institute, Meiji Yasuda Life Foundation of Health and Welfare, 150 Tobuki, Hachioji, Tokyo 192-0001, Japan; ta-jindo@my-zaidan.or.jp (T.J.);; 2 The Faculty of Human Life, Jumonji University, 2-1-28 Sugasawa, Niiza, Saitama 352-8510, Japan; 3 Okamura Corporation, Tenri Bldg, 1-4-1 Kitasaiwai, Nishi-ku, Yokohama, Kanagawa 220-0004, Japan; 4 Information Services International-Dentsu (ISID), LTD, 2-17-1 Konan, Minato-ku, Tokyo 108-0075, Japan

**Keywords:** office renovation, office layout, sit-stand desk, workplace health promotion, physical activity, sedentary behavior

## Abstract

It has been reported that office environment is an important determinant of physical activity (PA) and sedentary behavior (SB) in office workers. However, the effect of changes in office environment (office renovation) is unclear. The purpose of this study was to examine PA, SB, and space utilization changes among office workers in response to office renovation. This study was a natural experiment at three offices of a single company in Tokyo, Japan. The participants were, 13 workers from one office in the renovation group (mean age: 37.9 ± 10.8 years, percentage of females: 23.1%) and 29 from two offices in the control group (mean age: 42.3 ± 11.2 years, percentage of females: 31.0%). In the renovation, introduction of activity-based working (ABW) and installation of height-adjustable desks (HAD) were adopted. The ABW office was designed to provide various shared workstations, enabling the workers to choose workstations depending on their task or mood. Accelerometer measurement and object detection method using artificial intelligence (AI) technology for video images were used to assess behavior and space utilization before and after the renovation. Two weeks after the renovation, significant improvements in SB (pre- to post-renovation improvements: 346.8 ± 28.6 to 321.2 ± 17.8 min/working-hours) and PA (total PA: 173.2 ± 28.6 to 198.8 ± 17.8 min/working-hours; and light-intensity PA: 130.4 ± 27.1 to 150.7 ± 31.0 min/working-hours) were observed. In addition, the results of the object detection analysis showed that the central aisle of the office and shared HAD workstations near the entrance or window were utilized more frequently than the other spaces. This study suggested that office renovation could improve SB and PA immediately after the renovation. Moreover, utilized spaces and HAD workstations could play an important role to enhance employees’ activity in an ABW office.

## 1. Introduction

Previous studies have reported that a long duration of sedentary behavior (SB), which is defined as an activity intensity of 1.5 metabolic equivalents (METs) or lower [1], is associated with deterioration of physical and mental health [2,3], and also relates to low work engagement among workers [4,5]. It is therefore an emerging issue for workers to reduce the long duration of SB such as remaining seated during office work. For the management of a company to promote workers’ health and productivity, replacing sitting with standing or walking during working hours seems to be a feasible and effective countermeasure for this issue [6]. The implementation of change in office environment, such as layout of the office floor or desk type could be important measures of workers’ health and productivity. In the last decade, many studies have reported the effects of changes in office environment on SB during working hours [7]. Shrestha et al. [7] had conducted a systematic review to examine the effect of height-adjustable desks (HAD) on change in SB among employees during working hours and reported that the installation of HAD at the office could reduce the SB by over one hour per workday. Some other studies have also reported the relationship of office layout with SB among office workers [8,9]. However, research evidence related to the association between physical activity (PA) or SB and office layout are limited to only cross-sectional investigations, and hence, further study with longitudinal investigation on this issue is needed. 

In addition to these changes in the office environment, recent studies have suggested that introducing a new working style of “activity-based working (ABW)” could be a reasonable and effective option for management of PA and SB in companies and for employees [10,11]. ABW is a new working style that involves holistic approaches targeting behavioral (i.e., harnesses the intersection of the people), virtual (i.e., mobilization of information, knowledge sharing with technology), and physical (i.e., facilitating activities in various type of workstations) environments in workplaces [10,11]. The representative strategy for ABW is providing various shared workstations designed to intersperse in the office, enabling workers to choose workstations depending on their task or mood on a moment-to-moment basis. A systematic review of ABW [11] has reported that the implementation of ABW had positively impacted communication, control of time and space, and satisfaction with the workplace among office workers. 

There have been limited studies investigating the effect of ABW on physical activity (PA) or SB [12,13,14,15]. Most of the previous studies had limitations of study protocol (i.e., lack of control group), and one study had a control group but did not find significant results of the effect. Thus, further study is needed to confirm the effect of ABW on these behaviors. Another limitation in the earlier studies is the methodology for assessment of behavior at the office. An ABW office provides the opportunity for workers to work at various workstations depending on their situation. Since utilized spaces would be essential for an ABW office, and these spaces might contribute to reduce SB and increase PA, it should be clarified how often each ABW space would be utilized by the employees. These findings related to space utilization would be helpful to make effective plans and implement ABW renovation to enhance employees’ activity. However, an accelerometer, used most commonly for behavior measurement in previous studies, cannot evaluate which space and station at the office has been frequently or infrequently used through the office renovation. To solve this problem, another method that can evaluate the frequency of usage of a specific place by office workers should be adopted. Based on the rapid development of artificial intelligence (AI) technologies, a new behavior assessment method using this technology has been developed, which could be used for behavior science. This new method can immediately and accurately detect various objects, including people [16]. It might be possible, therefore, to evaluate exact changes in the utilization of office spaces by using this method. 

The primary purpose of this study was to investigate the changes in PA and SB among employees in response to office renovation with the adoption of ABW and HAD. In addition, this study aimed to identify the utilized space in the renovated office using the motion detection method driven by AI technology, wherein the effective method of office renovation to enhance employees’ activity would be clarified. We hypothesized that office renovations adopting ABW and HAD will replace SB with PA, similar to the installation of HAD [7]. Moreover, it is expected that the adoption of ABW will generate a utilization gap between each aisle and workstation.

## 2. Materials and Methods 

### 2.1. Study Design and Participants

This study was a natural experiment at three offices of a single company in Tokyo, Japan. The participants, 67 employees (26 from one office in the renovation group and 41 from two offices in the control group), were registered at the baseline of this study. All those who had worked at the target offices were included in the study, and those for whom data were missing for analysis and transfer to the other office during the study period were excluded. Participants who did not wear the accelerometer or did not have valid accelerometer data (13 participants in the renovation group and 12 in the control group) were excluded from the analysis. Thus, data from 13 participants in the renovation group (mean age: 37.9 ± 10.8 years, age range: 24–54) and 29 in the control group (mean age: 42.3 ± 11.2 years, age range: 23–67) were used in the analysis. 

As part of the ethical procedure, the office superiors explained the aim and procedure of the study to the employees using printed material provided by the researchers. The employees were asked to read the instructions of the study which explained how personal information would be used and managed, that the decision to participate should be made based on the free will of the individual, and that the results of the investigation would not be used for individual performance appraisal by the company. Every eligible participant provided informed consent to participate in this study. In addition, the research support personnel in the company explained the procedure of motion movie recording and its usage before recording, and every employee accepted the same. This study plan was approved by the Ethical Committee of Meiji Yasuda Life Foundation of Health and Welfare (no. 29001).

### 2.2. Study Protocol

**Figure 1:**
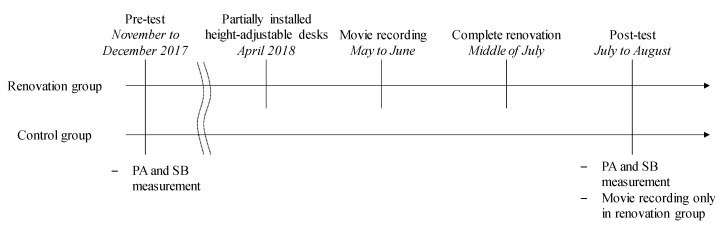
Flow of the study. PA: physical activity; SB: sedentary behavior.

The study protocol is shown in Figure 1. The baseline survey, including PA and SB measurements as the pre-test, was conducted in both the renovation and control groups from November 2017 to December 2017. The installation of HAD and the renovation of the office layout started in April 2018 in the office to be renovated and was completed in the middle of July 2018. The survey including PA and SB measurements for the post-test was conducted in both groups from July 2018 to August 2018. The motion video was recorded only in the renovation group between May 2018 and June 2018 for the pre-test and between July 2018 and August 2018 for the post-test. In the two control offices, no office renovation was conducted during the study period. 

### 2.3. Renovation Offices

#### 2.3.1. Pre-Renovation

**Figure 2:**
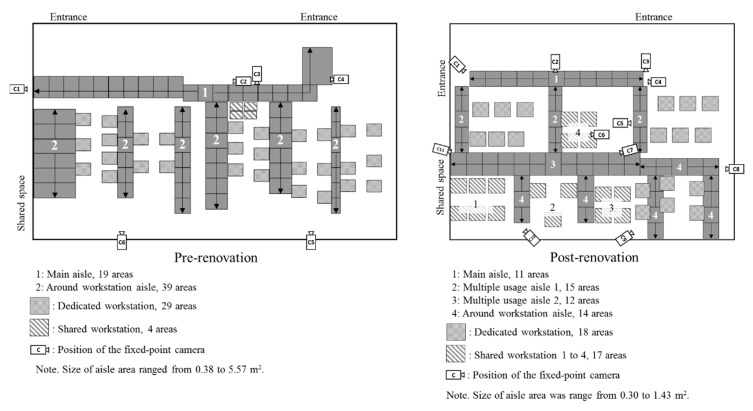
Aisle and workstation spaces on the office drawing at pre- and post-renovation.

The office drawings for the pre- and post-renovation periods are shown in Figure 2. The renovated office was a 371 square meters room on a floor of a shared use building. The shared space and meeting room on the left side of the office were not used in this study because of limited equipment for video recording. The office layout and desk arrangement at pre-renovation were in the traditional Japanese style (left in Figure 2), and the desks were all sit-type. Every employee was provided a dedicated workstation, and some shared workstations were placed in the workspace. HADs were introduced to some spaces in April 2017 prior to the complete renovation. The standard working hours for all employees were from 8:40 to 17:20, and the usual lunch break was from 12:00 to 13:00.

#### 2.3.2. Post-Renovation

The office renovation was completed in the middle of July 2018. The concept of renovation consisted of three components: “Fitness,” “Well-being,” and “Learning.” “Fitness” aimed to reduce SB and ensure that employees are working actively; “Well-being” aimed to enable understanding of other persons, including co-workers, thereby creating a human-friendly environment; and “Learning” aimed to facilitate interaction or communication with many people through learning experiences of mutual understanding. Based on the above concept, the environment changes at the office were conducted by adopting height-adjustable desks or tables for all workstations. Shared workstations were placed in accordance with the renovation plans, and 11 out of 29 dedicated workstations were changed to shared workstations. 

The dedicated workstations were used mainly by the office clerks, with some managers using them on occasion. The shared workstations were used by the workers in sales and service sections. The shared workstations were available for all employees and visitors to use. The office layout was changed drastically, and round aisles and various workstations were introduced. 

### 2.4. Control Office

Two control offices were in different shared buildings. One of the control offices was a 406 square meter room, and sit-desks were equipped for every dedicated workstation. The other office was a 191 square meter room, and half of the dedicated workstations were sit-desks and the rest were changed to HADs before the pre-test. There was no change in office layout or desk type during the follow-up period in both offices. The standard working hours and the usual lunch break were the same as in the office that was being renovated. 

### 2.5. Measures

#### 2.5.1. Physical Activity and Sedentary Behavior 

The procedures for measurement of PA and SB have been reported in detail in a previous study [17]. A triaxial-accelerometer with an epoch length of 60 s (Active style Pro HJA-750C; Omron Healthcare Co. Ltd., Kyoto, Japan) was used for the measurement of the activities in this study. The validity of the device for the measurement of PA and SB has been examined and reported in prior studies [18,19,20,21]. 

The accelerometer was distributed with measurement instructions and a questionnaire to each participant. The participants were instructed to wear the accelerometer at all times while they were awake for two weeks, except when swimming or bathing. “Non-wear” time was defined as an interval of at least 20 consecutive minutes under the detectable intensity of the accelerometer [22], and a valid day as a day when the participant had 10 or more hours of wearing time [23]. Since a previous study reported that a cut-off of 20 min for non-wear time showed the lowest misclassification (i.e., wear labelled as non-wear and non-wear labelled as wear) in adults compared to longer cut-offs such as 60 min [22], we decided to adopt the criterion in this study. The data from participants who had four or more valid days per working week were treated as valid data [24]. SB was defined as an activity with an intensity of less than or equal to 1.5 METs and PA (total) with an intensity of greater than or equal to 1.6 METs. In addition, PA was divided into light-intensity PA (LPA; 1.6–3.0 METs) and moderate- to vigorous-intensity PA (MVPA; ≥ 3.0 METs). Prolonged SB was defined as consecutive SB for 30 min or longer. All of these variables were calculated during the standard working hours (8:40–17:20) at the office and during all days on weekdays. To convert the data on a workday into the units of min/working hours per workday, the following formula was used: min/working hours = observed duration / wearing time × standard working hours (8.67 h).

#### 2.5.2. Video Recording and Analysis of the Video Data

To objectively assess the change in utilization of each space and workstation at the renovated office, the motion of the office worker was recorded using fixed-point cameras, and the motion videos were analyzed using AI technology. These procedures were conducted by the Information Services International-Dentsu, LTD. Go Pro Hero 5 (Go Pro Inc., San Mateo, California, USA), light action cameras set at full high definition (1920 × 1080), 60 films per second, and wide angle were used for video recording. The fixed points of the cameras are shown in Figure 2. Six cameras were used to record data before renovation and 11 cameras for after renovation. Video recording at the office was conducted between 10:00 and 17:00 for three days during pre- and post-renovation. Video data could not be obtained between 10:00 and 10:30 in the pre-renovation phase owing to machine trouble, and data of a single camera on day 2 in the post-renovation phase were not available because of the wrong angle of the camera.

For the analysis of video data, Darknet, which is a framework of deep neural networks, was used in this study. Darknet is one of the implementation libraries of Yolov3, which is a state-of-the-art, real-time object detection system [16,25], and these AI technologies are open source to the public [26]. Darknet was adopted for detection and recording of the number of “persons” as the target object. Each area was labeled for the applicable usage of space, including owned/dedicated or shared workstation, and main or around workstation aisles. Each space was defined as follows; the owned/dedicated workstation is available only for the assigned person, the shared workstation for all employees, the main aisle is used for transferring from and to the office destinations other than to directly access a workstation, around-workstation aisle is directly accessible to a workstation, and the multiple usage aisle is used for both transferring from and to the destinations and to directly access a workstation. In this study, the detectable number of persons in each space was defined as space utilization. The detailed procedures of the video analysis and its accuracy are shown in Appendix A and Appendix B. 

#### 2.5.3. Demographic Variables

Demographic variables including age, gender, body mass index (BMI), educational level, subjective economic status (very poor, poor, good, very good), and job type (manager, office clerk, service, or sales) were assessed by a self-reported questionnaire. 

In addition, participants were asked to record their entry and leaving time to the office during the pre- and post-test phases to confirm whether the time at the workplace had changed. If participants repeatedly entered and exited the office in a single day, they were asked to record all entry and exit times. Average time in office was calculated from these recorded data in each test period. 

### 2.6. Statistical Analysis

Descriptive statistics included the mean ± standard deviation for the continuous variable, and number and percentage for the categorical variable. Baseline characteristics of the participants were compared between the two groups using independent t-test or chi-square test. To reveal the changes in PA and SB from pre- to post-test, two-way repeated-measures analysis of variance with group (renovation and control group) × time (pre and post-test) was conducted. Bonferroni post hoc test was adopted if a significant interaction was observed.

In the video data analysis, group (each space) × time (28 time slots; 10:00 to 17:00, separated per 15 min) repeated-measures analysis of variance was conducted for pre- and post-renovation. Since area sizes in the aisle space were different between pre- and post-renovation, it was treated as the covariate in the analysis.

SPSS version 25.0 (IBM, Inc., Armonk, NY, USA) was used for the analysis. The level of statistical significance was set at *p* < 0.05.

## 3. Results

### 3.1. Individual Data Analysis

#### 3.1.1. Participants’ Characteristics 

Table 1 presents participants’ characteristics, PA, and SB in the two groups. There was significant difference in job type between the groups, while other variables did not show group differences at baseline. The control group showed a higher percentage of office clerks than the renovation group. There was a significant difference in MVPA in the all-day data for weekdays, and the renovation group showed higher amount of MVPA than the control group. Even though there was no significant baseline difference in SB time, the renovation group showed a shorter SB time compared with the control group. These differences in MVPA and SB seem to be due to the difference in job type between the groups. 

#### 3.1.2. Changes in PA and SB

Table 2 shows the changes in PA and SB from pre to post-renovation in the two groups. Regarding accelerometer data, the number of valid days (renovation: 8.9 ± 1.7 days, control: 8.4 ± 1.7 days), wearing time during working hours (renovation: 509.9 ± 11.4 min, control: 510.8 ± 14.1 min), and all-day wearing time on weekdays (renovation: 873.9 ± 55.1 min, control: 896.4 ± 102.7 min) were similar between the two groups in the post-test. Judging from the wearing time, misclassification of SB to non-wear time might be low because the average wearing time was almost the entire duration of working time (around 98%). In addition, wearing time seems to not affect the results because there were no differences between the two groups. There were significant interactions in SB, total PA, and LPA during working hours, and we observed significant improvements in these variables in the renovation group (pre- to post- improvements were: SB; 346.8 ± 28.6 to 321.2 ± 17.8 min/working-hours, total PA; 173.2 ± 28.6 to 198.8 ± 17.8 min/working-hours, and LPA; 130.4 ± 27.1 to 150.7 ± 31.0 min/working-hours). In the overall data in the working week, there were significant interactions in SB and total PA (changes were: SB; 569.0 ± 74.2 to 526.7 ± 56.4 min/day, and total PA; 312.5 ± 42.9 to 347.3 ± 43.5 min/day), but not in LPA. 

The average time and standard deviation of time in office in pre- and post-renovations were 6.2 ± 2.0 and 7.3 ± 5.0 h/day in the renovation group, and 7.2 ± 1.7 and 7.7 ± 2.2 h/day in the control group. There was a significant main effect of time without interaction in time in office (*p* = 0.038).

### 3.2. Movie Analysis for the Renovation Office

#### 3.2.1. Pre-Renovation

In the utilization of the aisle space (Table 3), there was a significant main effect of time, but no significant effect of the space and interaction. There was no difference in the detectable number of persons between main and around-workstation aisles (59.5 ± 6.8 and 63.9 ± 9.7 numbers/15 min, respectively). 

With the utilization of the workstation space, although the statistical analysis was not conducted because of limited number of shared workstations, most of the detections of persons were obtained at the dedicated workstations (1016.7 ± 151.7 numbers/15 min) compared to the shared workstation (241.1 ± 408.4 numbers/15 min). 

#### 3.2.2. Post-Renovation

In the utilization of the aisle space, there were significant main effects of space and time, but not of interaction. The main aisle and the multiple usage aisle 2 had larger detectable numbers than the multiple usage aisle 1. 

With the workstation space, there were significant interaction and main effects of space and time. The dedicated workstations showed larger detectable numbers than the shared workstation (1629.0 ± 184.0 and 442.3 ± 189.3 numbers/15 min, respectively), which was similar to the pre-renovation results. 

**Figure 3:**
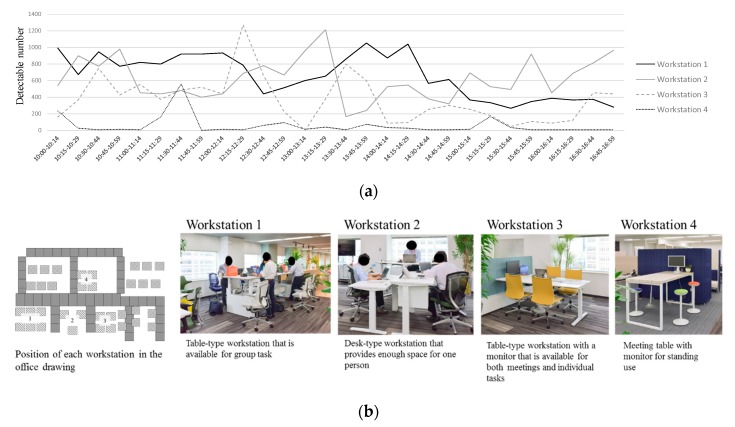
Detectable number of persons in each shared workstation at post-renovation. (**a**) detectable number in each time slot; (**b**) location and characteristics of each shared workstation.

Regarding the detection difference at each shared workstation space (Figure 3), there seemed to be some variations in its usage through working days. The timing of frequent and infrequent detection appeared to be different at each workstation. On the other hand, shared workstation 4, which can be used in standing position, had fewer persons detected than the other workstations.

## 4. Discussion

This study examined the changes in office activities and space utilization among employees in response to office renovation using not only accelerometer measurement but also video analysis with AI technology. As the result of ABW office renovation, including the installation of HADs, SB and PA were significantly improved. In addition, video analysis elucidated that the round-type aisle with a broad central aisle, dedicated workstations, and specific shared workstations in the ABW office were better utilized. These findings may contribute to effective office renovation to enhance employees’ activity by focusing on utilized space or HAD workstations. 

This study found significant improvement in SB and PA through the office renovation, and the strength of this study was the setting of the control group. Since most previous studies regarding ABW [13,14,15] were limited by the lack of a control group, this study added further knowledge about the effectiveness of ABW renovation. Results showed that, the change in overall SB by the renovation was around 25 min per 8.67 h workday or 40 min per weekday. Since the time in office did not decrease from pre to post-renovation, the reduction in overall SB may be due to an increase in PA, especially in LPA such as slow pace walking, standing, or changing posture during working hours. An earlier systematic review of interventions related to reduction in workplace SB [7] reported that the installation of HAD reduced overall SB by around 100 min per workday in a short-term follow-up (up to three months). Although the ABW office with HAD might have potential to further reduce SB, the effect was smaller than the effect with only installation of HAD. To reduce SB greatly in an ABW office, combining organizational, educational, or behavioral approaches [27,28] should be considered. A previous study [7] has also confirmed an improvement in prolonged SB lasting for more than 30 min owing to the introduction of HAD. The current study, however, did not show a significant change in prolonged SB in response to office renovation. A result similar to that of this study has been reported in another ABW study [15]. Although the result of this study might partly be due to the small sample size or baseline difference between the groups, it might also be possible that the effect of office renovation on the prolonged SB is different due to the difference in changes in the environment. The details of these results should be examined in a future study. 

In addition to the change in individual activities in response to office renovation, video analysis indicated significant changes in utilization of office space by the workers. The aisles at the office were changed to round-type aisles in the renovation phase, which created some space for multiple purposes, such as transferring from and to office destinations and direct access to a workstation. As a result of these changes, the main and central aisles (multiple usage aisle 2) were more frequently used by the workers in the post-renovation period. Although it is difficult to compare the specific time slot between pre- and post-renovation due to the difference in detection accuracy, overall detection of time slots for aisle use appears to have increased from pre to post-renovation. Accordingly, these results indicated that the round-type aisle with a broad central aisle is an essential element of office layout, which may contribute to an increase in PA among the workers. 

Although the number of shared workstations was increased in the post-renovation phase, the detected number of persons was larger at the dedicated workstations than at the shared workstations in both pre- and post-renovation phases. The reason for this might be the difference in the assigned job type for each workstation. Shared workstations were more likely to be used by employees who worked in sales and service, who tend to frequently stand up or go outside the office. On the other hand, there were specific characteristics of utilization in each shared workstation space on a moment-to-moment basis. Workstation 1 was constantly utilized throughout working hours, while workstations 2 and 3 were mainly utilized during the lunch break or in the afternoon. Workstation 4, however, had comparatively fewer detections than the other workstations. It is speculated that the location (center of the office) or the desk type (only for standing use) may be the reason for short or infrequent use. The different pattern of utilization between the shared workstations might be due to the differences in the desk type (i.e., number of seats, standing use, or sit–stand use) and the location of workstation (i.e., access from the entrance, near the window or center of the office). These results suggested that HAD workstations near the entrance or the window would be utilized, and accordingly, standing to work at these workstations could possibly contribute to a large reduction in SB. 

The results from this study indicate that, in the planning of office renovation, how to set aisles and workstations to enhance employee’ activity should be considered. In addition to the activity change, future studies should clarify how change in PA or SB owing to office renovation affects employees’ health, work productivity, communication, etc. These comprehensive effects could contribute to improving a company’s value (i.e., stock performance [29]) or reducing healthcare costs. 

This study has several limitations. First, as the accelerometer data were not obtained from every employee in the target offices, sampling bias would be present. For example, the conflicting results regarding the change in prolonged SB between a previous study introducing HAD [7] and the current study could be due to weak statistical power. In addition, there were differences in job type, MVPA, and SB (not significant) at baseline; therefore, the degree of change in PA and SB might be affected by differences other than the impact of office environment change. To obtain generalizable findings, a study with high comparability (i.e., cluster randomized controlled trial) and a larger sample size should be conducted. Second, the accelerometer used in this study has been reported to underestimate the duration of SB and overestimate breaks from prolonged SB compared to thigh-worn activPAL3 [21]; thus, direct comparisons with previous studies using different devices should be performed cautiously. Third, video analysis was conducted without identifying an individual participant owing to compliance with the ethical agreement, and hence the result of the accelerometer and the video analysis could not be combined. In addition, the data of the video analysis were not compared between the pre- and post-renovation phases because of the difference in accuracy of detection. These findings made it impossible to identify the individual changes in activities and space uses in response to office renovation. Finally, this study evaluated the activity change immediately after the renovation (around two weeks), and hence the long-term change or maintenance of the activities is unclear. The acute changes after the renovation could be the consequence of the “new effect” or curiosity. Unfortunately, the follow-up information, such as half or a year after the renovation, could not be obtained in this study because many participants were transferred to another office. Future study should be conducted for the examination of changes in the activities and space utilizations during a long-term follow-up of office renovation. If ethical approval can be obtained for linking the accelerometer with video analysis data, the reciprocal relationship between SB, PA, and space utilization could be clarified in a future study.

## 5. Conclusions

Office renovation including the introduction of ABW and HAD could improve PA and SB among office workers immediately after the renovation. Moreover, a round-type aisle with a broad central aisle, dedicated HAD workstation, and the shared HAD workstation near the entrance or the window were utilized in the ABW office. These utilized spaces and workstations could play an important role in enhancing employees’ activity.

## Figures and Tables

**Table 1:** Baseline characteristics in participants who wore triaxial-accelerometer.

Variables	Control	Renovation	*p*-value
	(n = 29)	(n = 13)	
	M ± SD	M ± SD	
	Number (%)	Number (%)	
Age, years old	42.3 ± 11.2	37.9±10.8	0.243
Gender			
Male	20 (69.0)	10 (76.9)	0.722
Female	9 (31.9)	3 (23.1)	
Body mass index, kg/m^2^	22.6 ± 4.1	21.7±3.7	0.555
Education, year	15.9 ± 0.9	15.8 ± 0.8	0.677
Subjective economic status			
Very poor	1(3.4)	0 (0.0)	0.742
Poor	8 (27.6)	3 (23.1)	
Good	20 (69.0)	10 (76.9)	
Very good	0 (0.0)	0 (0.0)	
Job type			
Manager	12 (41.4)	7 (53.8)	0.037
Office clerk	13 (44.8)	1 (7.7)	
Service or sales worker	4 (13.8)	5 (38.5)	
Physical activity and sedentary behavior			
Number of valid days	7.4 ±1.4	8.2±1.0	0.055
During standard working hours			
Wearing time, min/working hours	512.3±14.1	513.9±11.8	0.729
SB time, min/working hours	365 ± 42	346.8±28.6	0.164
Prolonged SB time, min/working hours	110.8 ± 68.4	95.3 ± 43.5	0.382
Total PA, min/working hours	155 ± 42	173.2 ± 28.6	0.164
Light-intensity PA, min/working hours	122.3 ± 36.4	130.4 ± 27.1	0.475
Moderate- to vigorous-intensity PA, min/working hours	32.7 ± 15.7	42.8 ± 15.9	0.063
All day in weekday			
Wearing time, min/day	895.1 ± 98.2	881.5 ± 63.7	0.65
SB time, min/day	592.8 ± 68.3	569 ± 74.2	0.317
Prolonged SB time, min/day	177.3 ± 86.6	158.9 ± 76.7	0.514
Total PA, min/day	302.4 ± 77	312.5 ± 42.9	0.662
Light-intensity PA, min/day	226.8 ± 69.8	224 ± 37.1	0.866
Moderate- to vigorous-intensity PA, min/day	75.6 ± 18.9	88.5 ± 18.4	0.046

SB: sedentary behavior, PA: physical activity, M: mean, SD: standard deviation; Note. Prolonged SB was defined as sitting time lasting for 30 min or longer. Working hours indicate the standard working hours of the company (i.e., 8.67 h; 520 min).

**Table 2:** Changes in sedentary behavior and physical activity from pre- to post-test in each group.

Variables	Pre-test	Post-test	Time Effect *p*-value	Interaction *p*-value	Post Hoc Test Pre- vs. Post-test *p*-value
	M ± SD	M ± SD			
During standard working hours					
SB time, min/working hours					
Control	365.0 ± 42.0	367.8 ± 48.1	0.028	0.007	0.618
Renovation	346.8 ± 28.6	321.2 ± 17.8			0.004
Prolonged SB time, min/working hours					
Control	110.8 ± 68.4	107.8 ± 60.9	0.188	0.340	
Renovation	95.3 ± 43.5	76.7 ± 42.0			
Total PA, min/working hours					
Control	155.0 ± 42.0	152.2 ± 48.1	0.028	0.007	0.618
Renovation	173.2 ± 28.6	198.8 ± 17.8			0.004
Light-intensity PA, min/working hours					
Control	122.3 ± 36.4	120.5 ± 34.7	0.045	0.018	0.723
Renovation	130.4 ± 27.1	150.7 ± 31.0			0.009
Moderate- to vigorous-intensity PA, min/working hours					
Control	32.7 ± 15.7	31.7 ± 18.4	0.331	0.155	
Renovation	42.8 ± 15.9	48.1 ± 21.2			
All day in weekday					
SB time, min/day					
Control	592.8 ± 68.3	597.4 ± 93.0	0.084	0.033	0.698
Renovation	569.0 ± 74.2	526.7 ± 56.4			0.022
Prolonged SB time, min/day					
Control	177.3 ± 86.6	179.3 ± 106.8	0.257	0.198	
Renovation	158.9 ± 76.7	127.4 ± 63.6			
Total PA, min/day					
Control	302.4 ± 77.0	299.0 ± 83.7	0.050	0.018	0.694
Renovation	312.5 ± 42.9	347.3 ± 43.5			0.010
Light-intensity PA, min/day					
Control	226.8 ± 69.8	223.6 ± 67.7	0.158	0.066	
Renovation	224.0 ± 37.1	247.6 ± 58.9			
Moderate- to vigorous-intensity PA, min/day					
Control	75.6 ± 18.9	75.4 ± 25.9	0.113	0.099	
Renovation	88.5 ± 18.4	99.6 ± 28.0			

SB: sedentary behavior, PA: physical activity, M: mean, SD: standard deviation; Note. Prolonged SB was defined as sitting time lasting for 30 min or longer. Working hours indicate the standard working hours of the company (i.e., 8.67 h; 520 min).

**Table 3:** Detectable number of persons in each space at pre- and post-renovation.

Variables	Detectable Number of Persons per 15 min	Time Effect *p*-value	Space Effect *p*-value	Interaction *p*-value
	M ± SE			
Pre-renovation				
Aisle				
Main aisle	59.5 ± 6.8	0.002	0.712	0.222
Around-workstation aisle	63.9 ± 9.7			
Workstation				
Dedicated workstation	1016.7 ± 151.7	Statistical analysis was not applicable		
Shared workstation	241.1 ± 408.4			
Post-renovation				
Aisle				
Main aisle	107.0 ± 15.0	< 0.001	< 0.001	0.246
Multiple usage aisle 1	27.6 ± 12.1			
Multiple usage aisle 2	135.2 ± 18.6			
Around-workstation aisle	57.2 ± 14.6			
Workstation				
Dedicated workstation	1629.0 ± 184.0	< 0.001	< 0.001	< 0.001
Shared workstation	442.3 ± 189.3			

M: mean, SD: standard error.

## Appendix B. Procedures for accuracy evaluation of the movie analysis and the results

A previous study reported that the Yolov3 could detect the object faster than the other detection methods with comparable accuracy (around 60 % average precision) (Redmon and Farhadi, 2018). However, there was no reference information of accuracy of video analysis in the practical office environment. We, therefore, evaluated the accuracy for the object detection in each camera at the pre and post-renovation. The pictures during 10:00–11:00 in the first day were used for accuracy evaluation. These pictures were randomly ordered, and research staff chose 50 pictures for each person, which were taken in either standing or sitting position. These pictures were analyzed using the Darknet for the calculation of the rate of false detection and leakage.

**Table A1:** Accuracy of each camera for detecting sitting or standing persons.

Cameras	Pre-renovation								Post-renovation							
	Sitting Person			Standing Person			False Detection per all Detection (%)		Sitting Person			Standing Person			False Detection per all Detection (%)	
	Detection	Actual	Leakage (%)	Detection	Actual	Leakage (%)			Detection	Actual	Leakage (%)	Detection	Actual	Leakage (%)		
Overall accuracy	671	1131	40.7	307	404	24.0	11	1.1	1019	1412	27.8	627	662	5.3	40	2.4
Camera 1	82	88	6.8	51	52	1.9	2	1.5	61	71	14.1	64	69	7.2	0	0.0
Camera 2	0	0	-	50	53	5.7	0	0.0	49	50	2.0	51	51	0.0	0	0.0
Camera 3	117	179	34.6	58	73	20.5	0	0.0	0	0	-	50	53	5.7	0	0.0
Camera 4	0	0	-	46	84	45.2	0	0.0	0	0	-	46	48	4.2	0	0.0
Camera 5	390	632	38.3	55	59	6.8	1	0.2	262	339	22.7	91	94	3.2	1	0.3
Camera 6	82	232	64.7	47	83	43.4	8	6.2	49	50	2.0	49	49	0.0	0	0.0
Camera 7	Not applicable								88	169	47.9	49	52	5.8	0	0.0
Camera 8									129	148	12.8	47	51	7.8	2	1.1
Camera 9									148	198	25.3	72	73	1.4	0	0.0
Camera 10									233	387	39.8	40	52	23.1	28	10.3
Camera 11									0	0	-	68	70	2.9	9	13.2

As the results of accuracy evaluation, rate of false detection was 1.1% (0.0–6.2%), and detection of leakage was 40.7% (6.8–64.7%) for sitting persons and 24.0% (1.9–45.2%) for standing persons in each camera in the pre-renovation. In the post-renovation test, the rate of false detection was 2.4% (0.0–13.2%), and detection of leakage was 27.8% (2.0–47.9%) for sitting persons and 5.3% (0.0–23.1%) for standing persons in each camera.

## References

[1] Bames J., Behrens T.K., Benden M.E., Biddle S., Bond D., Brassard P., Brown H., Carr L., Carson V., Chaput J. (2012). Letter to the Editor: Standardized use of the terms “sedentary” and “sedentary behaviours”. Appl. Physiol. Nutr. Metab..

[2] Patterson R., McNamara E., Tainio M., de Sá T.H., Smith A.D., Sharp S.J., Edwards P., Woodcock J., Brage S., Wijndaele K. (2018). Sedentary behaviour and risk of all-cause, cardiovascular and cancer mortality, and incident type 2 diabetes: A systematic review and dose response meta-analysis. Eur. J. Epidemiol..

[3] Zhai L., Zhang Y., Zhang D. (2015). Sedentary behaviour and the risk of depression: A meta-analysis. Br. J. Sports Med..

[4] Munir F., Houdmont J., Clemes S., Wilson K., Kerr R., Addley K. (2015). Work engagement and its association with occupational sitting time: Results from the Stormont study. BMC Public Health.

[5] Ishii K., Shibata A., Oka K. (2018). Work Engagement, Productivity, and Self-reported Work-related Sedentary Behavior among Japanese Adults: A Cross-sectional Study. J. Occup. Environ. Med..

[6] Edwardson C.L., Yates T., Biddle S.J.H., Davies M.J., Dunstan D.W., Esliger D.W., Gray L.J., Jackson B., O’Connell S.E., Waheed G. (2018). Effectiveness of the Stand More AT (SMArT) Work intervention: Cluster randomised controlled trial. BMJ.

[7] Shrestha N., Kukkonen-Harjula K.T., Verbeek J.H., Ijaz S., Hermans V., Pedisic Z. (2018). Workplace interventions for reducing sitting at work. Cochrane Database Syst. Rev..

[8] Fisher A., Ucci M., Smith L., Sawyer A., Spinney R., Konstantatou M., Marmot A. (2018). Associations between the Objectively Measured Office Environment and Workplace Step Count and Sitting Time: Cross-Sectional Analyses from the Active Buildings Study. Int. J. Environ. Res. Public Health.

[9] Lindberg C.M., Srinivasan K., Gilligan B., Razjouyan J., Lee H., Najafi B., Canada K.J., Mehl M.R., Currim F., Ram S. (2018). Effects of office workstation type on physical activity and stress. Occup. Environ. Med..

[10] Veldhoen+Company Workplace Trends 2012, Activity Based Working. https://www.slideshare.net/maggieprocopi/workplace-trends-2012-activity-based-working-in-the-netherlandslouis-lhoest.

[11] Engelen L., Chau J., Young S., Mackey M., Jeyapalan D., Bauman A. (2019). Is activity-based working impacting health, work performance and perceptions? A systematic review. Build. Res. Inf..

[12] Arundell L., Sudholz B., Teychenne M., Salmon J., Hayward B., Healy G.N., Timperio A. (2018). The impact of activity based working (ABW) on workplace activity, eating behaviours, productivity, and satisfaction. Int. J. Environ. Res. Public Health.

[13] Foley B., Engelen L., Gale J., Bauman A., Mackey M. (2016). Sedentary behavior and musculoskeletal discomfort are reduced when office workers trial an activity-based work environment. J. Occup. Environ. Med..

[14] Gorman E., Ashe M.C., Dunstan D.W., Hanson H.M., Madden K., Winkler E.A., McKay H.A., Healy G.N. (2013). Does an ‘activity-permissive’workplace change office workers’ sitting and activity time?. PloS ONE.

[15] Jancey J.M., McGann S., Creagh R., Blackford K.D., Howat P., Tye M. (2016). Workplace building design and office-based workers’ activity: A study of a natural experiment. Aust. N. Z. J. Public Health.

[16] Redmon J., Farhadi A. YOLO9000: Better, faster, stronger. Proceedings of the IEEE Conference on Computer Vision and Pattern Recognition.

[17] Jindo T., Makishima M., Kitano N., Wakaba K., Kai Y. (2019). Association of the usage of height-adjustable desks with physical activity and sitting behavior in employees. Bull. Phys. Fit. Res. Inst..

[18] Murakami H., Kawakami R., Nakae S., Nakata Y., Ishikawa-Takata K., Tanaka S., Miyachi M. (2016). Accuracy of wearable devices for estimating total energy expenditure: Comparison with metabolic chamber and doubly labeled water method. JAMA Intern. Med..

[19] Murakami H., Kawakami R., Nakae S., Yamada Y., Nakata Y., Ohkawara K., Sasai H., Ishikawa-Takata K., Tanaka S., Miyachi M. (2019). Accuracy of 12 Wearable Devices for Estimating Physical Activity Energy Expenditure Using a Metabolic Chamber and the Doubly Labeled Water Method: Validation Study. JMIR Mhealth Uhealth.

[20] Ohkawara K., Oshima Y., Hikihara Y., Ishikawa-Takata K., Tabata I., Tanaka S. (2011). Real-time estimation of daily physical activity intensity by a triaxial accelerometer and a gravity-removal classification algorithm. Br. J. Nutr..

[21] Kurita S., Yano S., Ishii K., Shibata A., Sasai H., Nakata Y., Fukushima N., Inoue S., Tanaka S., Sugiyama T. (2017). Comparability of activity monitors used in Asian and Western-country studies for assessing free-living sedentary behaviour. PLoS ONE.

[22] Peeters G., van Gellecum Y., Ryde G., Farias N.A., Brown W.J. (2013). Is the pain of activity log-books worth the gain in precision when distinguishing wear and non-wear time for tri-axial accelerometers?. J. Sci. Med. Sport.

[23] Masse L.C., Fuemmeler B.F., Anderson C.B., Matthews C.E., Trost S.G., Catellier D.J., Treuth M. (2005). Accelerometer data reduction: A comparison of four reduction algorithms on select outcome variables. Med. Sci. Sports Exerc..

[24] Trost S.G., McIver K.L., Pate R.R. (2005). Conducting accelerometer-based activity assessments in field-based research. Med. Sci. Sports Exerc..

[25] Redmon J., Farhadi A. (2018). Yolov3: An incremental improvement. arXiv.

[26] Darknet. https://github.com/pjreddie/darknet.

[27] Chu A.H.Y., Ng S.H.X., Tan C.S., Win A.M., Koh D., Müller-Riemenschneider F. (2016). A systematic review and meta-analysis of workplace intervention strategies to reduce sedentary time in white-collar workers. Obes. Rev..

[28] Taylor W.C., Suminski R.R., Das B.M., Paxton R.J., Craig D.W. (2018). Organizational Culture and Implications for Workplace Interventions to Reduce Sitting Time Among Office-Based Workers: A Systematic Review. Front. Public Health.

[29] Goetzel R.Z., Fabius R., Fabius D., Roemer E.C., Thornton N., Kelly R.K., Pelletier K.R. (2016). The Stock Performance of C. Everett Koop Award Winners Compared With the Standard & Poor’s 500 Index. J. Occup. Environ. Med..

